# Supplementary material for: Correlation between PD-L1 Expression of Non-Small Cell Lung Cancer and Data from IVIM-DWI Acquired during Magnetic Resonance of the Thorax: Preliminary Results
Source: Cancers (Basel). 2022 Nov 16;14(22):5634. doi: 10.3390/cancers14225634 (PMC9688282; doi:10.3390/cancers14225634)
Supplement: Supplementary file 1 [file cancers-14-05634-s001.zip › cancers-1999580-supplementary.pdf]

Supplementary Materials

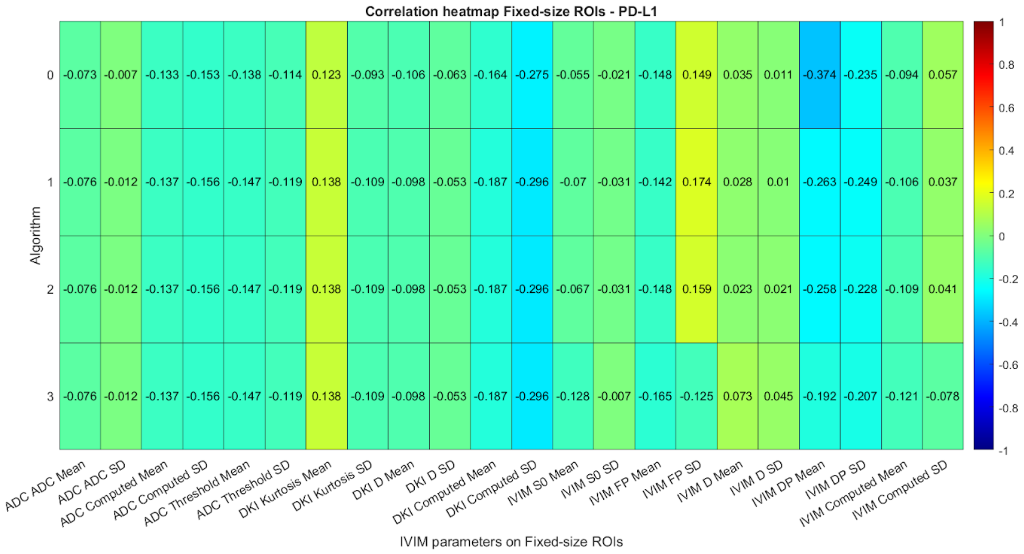

Figure S1. Correlation heatmap for fixed-size ROIs between the 22 IVIM-DWI parameters and the expression of PD-L1. Each row represents a different algorithm. Analysis on 19 patients.

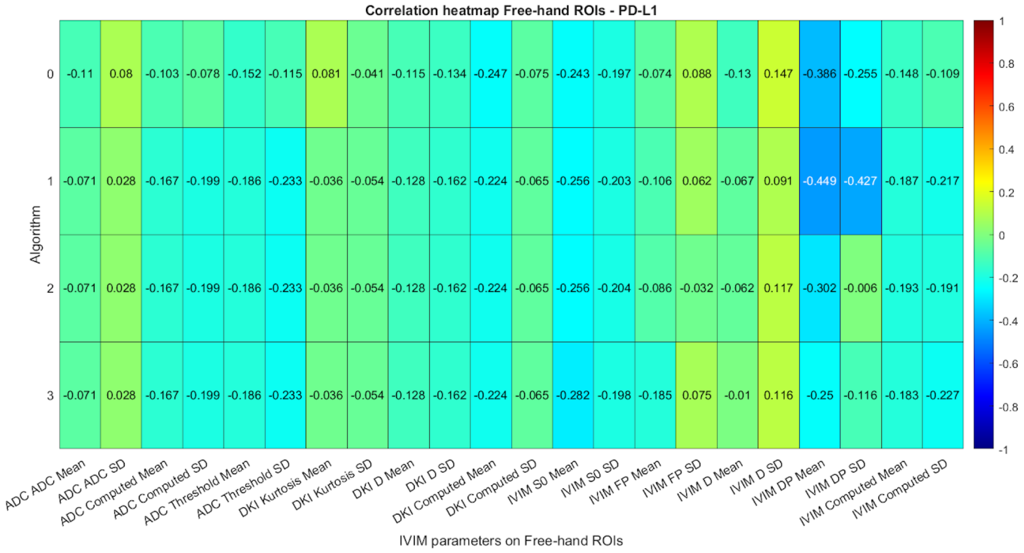

Figure S2. Correlation heatmap for freehand ROIs between the 22 IVIM-DWI parameters and the expression of PD-L1. Each row represents a different algorithm. Analysis on 21 patients.

Table S1. Table with results for fixed-size ROIs for algorithms 0, 1, 2 and 3. G0 refers to non-expression PD-L1 and G1 to positive expression of PD-L1.

Results for fixed-size ROIs						
	**Units**	**Mean**		**St.Dev.**		**P-val WMW**
		**G0**	**G1**	**G0**	**G1**	
Algorithm 0						
ADC	μm²/s × 10^6	1445.8	1519.8	359.6	310.7	0.842
D	μm²/s × 10^6	1173.3	1327.0	289.5	373.0	0.356
D*	μm²/s × 10^4	189.2	122.0	65.7	31.3	**0.008**
pf	μm²/s × 10^3	274.3	209.7	184.9	82.1	0.549
Algorithm 1						
ADC	μm²/s × 10^6	1447.9	1519.8	357.4	310.7	0.842
D	μm²/s × 10^6	1180.3	1331.8	286.9	370.0	0.352
D*	μm²/s × 10^4	317.8	199.0	133.0	83.2	0.065
pf	μm²/s × 10^3	276.6	216.9	185.9	86.2	0.497
Algorithm 2						
ADC	μm²/s × 10^6	1447.9	1519.8	357.4	310.7	0.842
D	μm²/s × 10^6	1189.2	1325.3	297.1	377.1	0.400
D*	μm²/s × 10^4	76.9	45.1	70.9	16.7	0.549
pf	μm²/s × 10^3	269.8	203.5	188.9	83.9	0.447
Algorithm 3						
ADC	μm²/s × 10^6	1447.9	1519.8	357.4	310.7	0.842
D	μm²/s × 10^6	1265.5	1499.4	434.9	378.0	0.156
D*	μm²/s × 10^4	295.6	213.0	149.0	118.1	0.243
pf	μm²/s × 10^3	325.6	241.9	187.8	61.9	0.661

Table S2. Table with results for free-hand ROIs for algorithms 0, 1, 2 and 3. G0 refers to non-expression PD-L1 and G1 to positive expression of PD-L1.

Results for free-hand ROIs						
	**Units**	**Mean**		**St.Dev.**		**P-val WMW**
		**G0**	**G1**	**G0**	**G1**	
Algorithm 0						
ADC	μm²/s × 10^6	1495.5	1630.8	334.8	395.1	0.594
D	μm²/s × 10^6	1245.5	1360.6	348.8	368.5	0.804
D*	μm²/s × 10^4	174.8	139.6	79.1	33.1	0.145
pf	μm²/s × 10^3	266.8	264.1	183.1	136.6	0.804
Algorithm 1						
ADC	μm²/s × 10^6	1458.4	1630.8	354.0	395.1	0.337
D	μm²/s × 10^6	1191.1	1365.0	356.9	366.4	0.456
D*	μm²/s × 10^4	286.1	210.8	108.5	60.6	0.127
pf	μm²/s × 10^3	287.6	270.2	172.3	136.6	0.915
Algorithm 2						
ADC	μm²/s × 10^6	1458.4	1630.8	354.0	395.1	0.337
D	μm²/s × 10^6	1201.1	1359.5	358.8	361.8	0.499
D*	μm²/s × 10^4	100.6	64.6	102.0	35.2	0.972
pf	μm²/s × 10^3	279.8	262.3	175.1	145.7	1.000
Algorithm 3						
ADC	μm²/s × 10^6	1458.4	1630.8	353.9	395.1	0.337
D	μm²/s × 10^6	1310.7	1556.8	561.9	404.8	0.145
D*	μm²/s × 10^4	263.6	228.9	144.3	61.3	1.000
pf	μm²/s × 10^3	368.2	304.1	189.1	115.9	0.594
